# Temperature Effect on Fracture of a Zr-Based Bulk Metallic Glass

**DOI:** 10.3390/ma13102391

**Published:** 2020-05-22

**Authors:** Na Yang, Jun Yi, Yu Hang Yang, Bo Huang, Yan Dong Jia, Sheng Zhong Kou, Gang Wang

**Affiliations:** 1 School of Materials Science and Engineering, Lanzhou University of Technology, Lanzhou 730050, China; Yn941129@163.com (N.Y.); koushengzhong@163.com (S.Z.K.); 2 Institute of Materials, School of Materials Science and Engineering, Shanghai University, Shanghai 200444, China; yangyuhang@shu.edu.cn (Y.H.Y.); huangb@shu.edu.cn (B.H.); yandongjia@shu.edu.cn (Y.D.J.); g.wang@shu.edu.cn (G.W.)

**Keywords:** bulk metallic glass, toughness, relaxation, shear band

## Abstract

Bulk metallic glass (BMGs) is highly expected for applications in engineering structures due to their superior mechanical properties. The fracture toughness of some BMGs was investigated at cryogenic and at elevated temperatures. However, the mechanism of the temperature-dependence of BMG toughness still remains elusive. Here, we characterized the fracture toughness of Zr_61_Ti_2_Cu_25_Al_12_ BMG prepared with Zr elemental pieces with low Hf content at temperatures ranging from 134 to 623 K. The relaxation spectrum of the BMG was characterized by a dynamic mechanical analysis using the same temperature range. We found that the BMG is tougher at onset temperatures of the relaxation processes than at peak temperatures. The temperature-dependent fracture toughness of the BMG is strongly dependent on its relaxation spectrum.

## 1. Introduction

Due to high strength [1,2,3], high toughness [4,5,6], and high fatigue resistance [7,8], bulk metallic glasses (BMGs) have potential applications as structural engineering materials. Usually, structural materials function at various temperatures. Temperature effects on mechanical properties of BMGs have been intensively investigated [9,10,11], and because thermal activation increases with increasing temperature, yield strength and hardness of BMGs decrease monotonically with increasing temperature [12,13,14,15,16,17]. The temperature dependence of plasticity [18,19,20] and toughness [21] are more complicated. There is brittle-to-ductile transition at cryogenic temperature [19] and intermediate temperature brittleness [18,21] as in crystalline metals [22,23]. The brittle-to-ductile transition of Ti6Al4V alloy is caused by oxygen [24] and the intermediate temperature brittleness occurs due to a transition from intra-grain plasticity to grain boundary failure in many crystalline metals [25]. However, the mechanism of the temperature-dependent toughness of BMGs remains elusive [21].

The fracture toughness of materials strongly depends on the flow units ahead of the crack tip [26]. Flow units in crystalline metals, such as dislocation, twining, and so on, can be observed using a transmission microscope [27] and theoretically described using physical models [28,29], which revealed the mechanism of temperature-dependent toughness of crystalline metals. The flow units in BMGs can be neither visualized experimentally nor physically modeled perfectly [30,31]. As such, the behavior of BMG flow units needs to be investigated to understand the temperature dependence of BMG toughness. 

Many research groups found that flow units correlated with peaks in relaxation spectrum of BMGs. Harmon [32] theoretically identified elastic deformation and plastic deformation with slow *β-* and *α*-relaxation, respectively, and stated that isolated STZ (shear transformation zone) and shear banding are associated with slow *β-* and *α*-relaxation, respectively. Dynamic mechanical analysis (DMA) of BMGs verified that activation of slow *β*-relaxation is nearly equal to the potential energy barriers of STZs [33], whereas compression tests showed that viscosity of shear band is quite similar to that of supercooled liquid, which undergoes *α*-relaxation [34,35]. It was reported [19] that the plasticity of BMGs at cryogenic temperature is mediated by another kind of flow unit that is associated with fast *β*-relaxation [36]. Therefore, we think that investigation of the correlation of different relaxation processes and flow units could shed light on the mechanism of the temperature-dependent fracture of BMGs.

Here, Zr_61_Ti_2_Cu_25_Al_12_ BMG with benchmark toughness was chosen for investigating the temperature effect on fracture toughness of the BMG. DMA was used to characterize the relaxation processes of the BMG. The toughness of the BMG was tested from cryogenic temperature to elevated temperature. Fracture morphology was also imaged to obtain detailed information about the fracture behavior of the BMG at various temperatures.

## 2. Materials and Methods 

Zr_61_Ti_2_Cu_25_Al_12_ alloy ingots were prepared by arc melting high purity Zr (≥99.95%) elemental pieces with low Hf content (≤0.03%), Ti (≥99.9%), Cu (≥99.9%), and Al (≥99.9%) elemental pieces in a Ti-gettered pure argon atmosphere in a vacuum chamber. Each alloy ingot was re-melted four times to ensure homogeneous composition. The Zr_61_Ti_2_Cu_25_Al_12_ plates with dimensions of 90 × 10 × 3 mm^3^ were fabricated by arc melting the ingots and a water-cooled copper mold suction casting.

Samples for fracture toughness tests with dimensions of B (thickness) × W (width) × L (length) = 3 × 6 × 30 mm^3^ were cut from the bottom of the as-cast plates using the electrical-discharge cutting technique and manual grinding with SiC papers. A straight-through notch with a root radius of 200 μm and a length of about 0.25 W was cut in the sample by using a diamond wire saw (Well 3500 Premium Version, Mannheim, Germany). Fatigue pre-cracking was performed on an Instron 8801 Servohydraulic Dynamic Testing System (Norwood, MA, USA) with a span (S) of 24 mm at a frequency of 20 Hz at ambient temperature. Fatigue pre-cracking was controlled by da/dN (crack propagation rate) fatigue crack propagation software (FastTrack 2) under decreasing stress intensity factor range (ΔK) mode with a constant stress ratio (R = P_min_/P_max_) of 0.1. The initial ΔK was 11 MPa⋅m^1/2^, which decreased with a normalized K gradient of −0.224. The end stress intensity factor at the tip of the fatigue pre-crack was around 7.5 MPa⋅m^1/2^. The total length of notch and fatigue pre-crack were about 0.45–0.5 W (confirming ASTM standard) after about 26,000 fatigue cycles. 

Fracture toughness tests at different temperatures were conducted in an environmental box with laboratory air on an Instron 8801 universal mechanical testing machine (Norwood, MA, USA) with a three-point bending fixture at a displacement rate of 0.1 mm/min. A clip gauge was used to monitor crack opening displacement (COD) across the crack mouth. The high temperatures were provided by a resistance heating wire and the low temperatures were supplied by inputting liquid nitrogen into the environmental box. After reaching the set temperature, 10 minutes were used for heat preservation to stabilize the temperature and subsequent fracture toughness tests. During the tests, the temperature fluctuation was within ±1 K. Three toughness tests were performed at each temperature to ensure repeatability of experimental results.

We investigated the relaxation behavior of Zr_61_Ti_2_Cu_25_Al_12_ glass using dynamic mechanical analysis (DMA) to determine the test temperatures for fracture toughness. The sample for DMA measurement, with a dimension of 30 × 2 × 1 mm^3^, was taken from the as-cast plate by using a diamond wafering blade on a low speed saw and subsequent grinding on SiC papers. DMA measurements were recorded on a TA Q800 DMA (New Castle, DE, USA) with a constant heating rate of 2 K/min at a frequency of 0.5 Hz. Finally, the morphology on side surface and fracture surface of the failed samples were observed in a Hitachi SU-1510 scanning electron microscope (SEM, Tokyo, Japan).

## 3. Results

### 3.1. Dynamic Mechanical Analysis 

**Figure 1:**
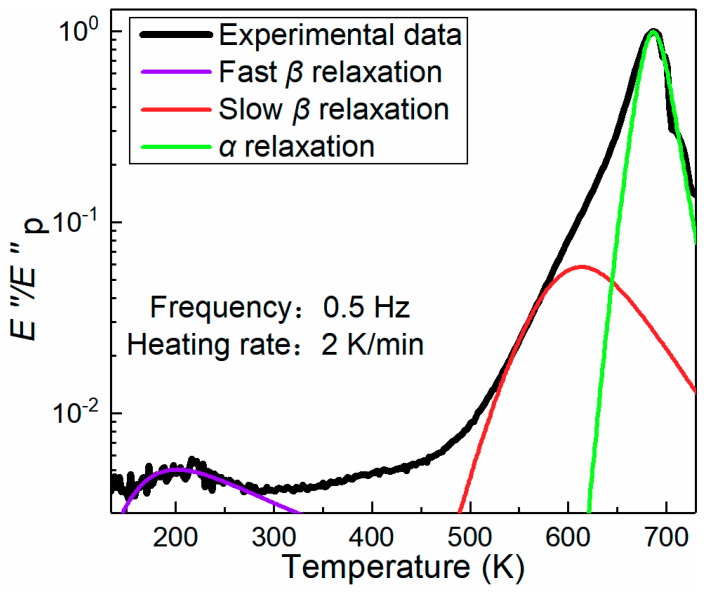
DMA (dynamic mechanical analysis) relaxation spectrum of the BMG (bulk metallic glass). The variation of loss modulus *E*″ with temperature were obtained at the frequency of 0.5 Hz and heating rate of 2 K/min. The black line indicates experimental data. The purple, red, and green curves represent the H-N (Havriliak-Negami) fitting of fast *β*-relaxation, slow *β*-relaxation, and *α*-relaxation, respectively (see details of the fittings in [36]).

The DMA relaxation spectrum of the BMG, obtained by measuring loss modulus *E*″ at temperatures ranging from 134 to 730 K, is shown in Figure 1. The *E*″ curve shows two distinct peaks in the temperature range: the peak temperature of 687 K is associated with *α*-relaxation and the peak at 207 K correlates to fast *β*-relaxation. In addition, a hidden peak is present, as a shoulder in the *α*-relaxation peak. This kind of hidden peak was identified as slow *β*-relaxation [37] with a peak temperature of 578 K. The onset temperatures of the fast *β*-relaxation and *α*-relaxation are 134 and 623 K in Figure 1, respectively. Between the slow and fast *β*-relaxation, there was a cage dynamic process that was thought to be the precursor of the slow *β*-relaxation, as reported in the literature [31]. Therefore, the onset temperature of the slow *β*-relaxation should be the inflection point, which was at 373 K. These characteristic temperatures and room temperature of 298 K were chosen for toughness tests of the BMG samples to see if there were some correlations between fracture toughness and the relaxation processes.

### 3.2. Toughness Test 

**Figure 2:**
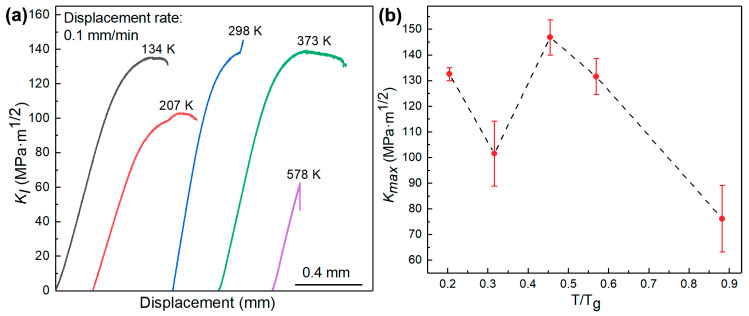
Temperature-dependent fracture toughness of the BMG samples. (**a**) K_I_ (mode I stress intensity factor) vs. crosshead displacement curve at various temperatures. (**b**) Fracture toughness of the samples at various temperatures.

The mode I stress intensity factor K_I_ vs. crosshead displacement curves are shown in Figure 2a. Except for the curve at 578 K, all other curves were nonlinear. The nonlinearity indicates plastic events ahead of fatigue crack tip. The plastic events were examined and will be shown in the next section. All these curves are typical for BMGs as reported in the literature [10,12]. A fracture toughness test was also performed at 623 K, however, the sample did not fracture. Hence, the toughness of the sample tested at 623 K could not be calculated. The temperature-dependent fracture toughness K_max_ of the BMG samples are shown in Figure 2b. From room temperature to elevated temperature, the dependence was similar to the reported intermediate temperature toughness minimum [21], and also similar to the reported intermediate temperature plasticity minimum [18]. However, the temperature-dependence of K_max_ of the BMG samples below room temperature was quite different from that of plasticity reported in the literature [18]. 

From Figure 1 and Figure 2b, the toughness at the onset temperature of relaxations was higher than the toughness at peak temperature of the relaxation processes. We think that at peak temperature of relaxation processes, the associated flow units were thermally activated and plasticity seldomly had flow units to mediate. We also think that at the onset temperature of relaxations, shear stress can activate associated flow units to undergo plastic deformation ahead of fatigue crack tip to increase fracture toughness of BMGs. Therefore, the BMG samples had higher toughness at 134, 298, and 373 K, whereas they had the lowest toughness at 207 and 578 K. The toughness values of the samples tested at these temperatures were 133, 102, 147, 132, and 76 MPa⋅m^1/2^, respectively.

**Figure 3:**
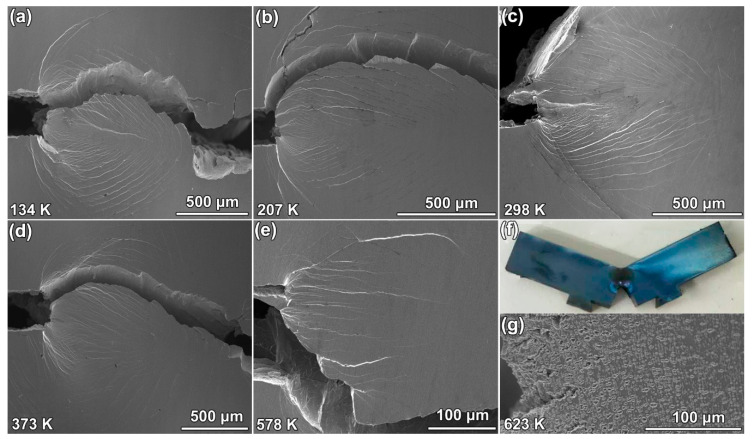
Side surface morphology of fractured specimens (**a**–**e**) fractured at 134, 207, 298, 373, and 578 K, respectively. The wide cuts on the left-hand side of the images are tips of fatigue pre-cracks. (**f**) The unfractured sample tested at 623 K. The blue color on the surface indicates oxidization. (**g**) SEM image of the region ahead of crack tip of the unfractured sample. A large void can be seen in the image.

K_max_ is an effective parameter used to compare fracture toughness of samples [5] tested at different temperatures. Due to the limited glass forming ability of the BMG, we could not obtain thicker samples to measure K_IC_. K_JC_ could not be precisely measured because there was crack deflection, as shown in the SEM images in Figure 3, and the standard K_JC_ test [28] supposed that crack propagates straight in the direction of fatigue pre-crack.

### 3.3. Plain-Stress Plastic Zone

To further investigate the temperature-dependent fracture behavior of the BMG samples, SEM was used to document the side surface fracture morphology of the samples fractured at various temperatures, as shown in Figure 3. Except the sample tested at 623 K, the samples underwent shear banding ahead of the fatigue crack tip. Samples fractured below room temperature had a fan-shaped plastic zone, as shown in Figure 3a,b. The plastic zone of the sample fractured at room temperature had the largest plastic zone (Figure 3c) extended from the fatigue pre-crack to the other end of the sample. Figure 3d,e show that there were fewer shear bands ahead of fatigue pre-crack in the samples tested at 373 and 578 K, respectively. The specimen tested at 623 K did not fracture, as shown in Figure 3f. No shear band was found ahead of the fatigue pre-crack and the material ahead of the crack tip underwent homogeneous plastic deformation, as shown. The fatigue pre-crack tip was strongly blunted. The blue surface indicated that the sample was oxidized during the toughness test. Figure 3g shows details of the oxidization. There were high-density voids around the crack tip. The voids might have been caused by failure of the interface between the oxide particles and the BMG matrix.

### 3.4. Fracture Pattern

**Figure 4:**
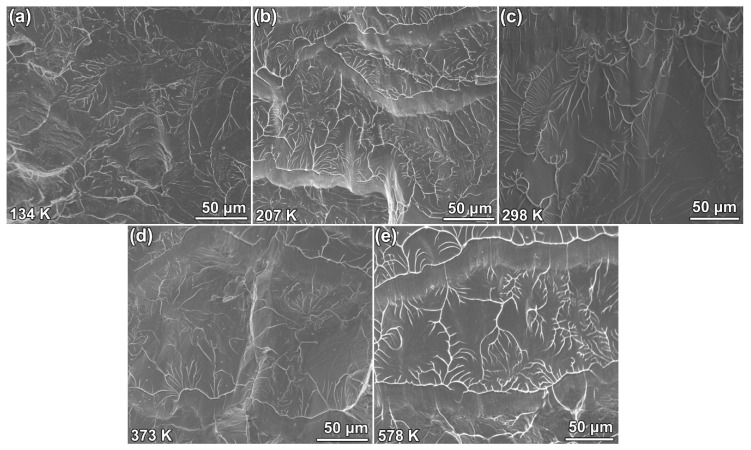
Pattern on the fracture surface of specimens tested at 134 (**a**), 207 (**b**), 298 (**c**), 373 (**d**), and 578 K (**e**).

As shown in Figure 4, no nano-corrugation was found on the fracture surface of specimens tested at all temperatures. There were dimples and viscous vein patterns on the fracture surface of the samples tested at 134 K, whereas the fractured samples tested at room temperature showed a viscous vein pattern that was similar to the pattern on the fracture surface of Zr_52.5_Cu_17.9_Ni_14.6_Al_10_ BMG fractured under pure shear [38]. The combination of viscous veins and shear sliding zones on the fracture surface of specimens, tested at 207, 373, and 578 K, were quite similar to the fracture pattern on the fracture surface of pre-compressed BMGs [38]. The viscous vein pattern corresponded to the sliding crack along the shear band and the shear sliding zone correlated with the deviation of crack from one shear band to another [39]. Because the deviation drives the crack toward the orientation of maximum hydrostatic tension in the plastic zone [39], it indicated domination of the maximum hydrostatic stress. Hence, the viscous vein pattern alone existed on the fracture surface of specimens, which was tougher than the samples with the combination of viscous vein pattern and the shear sliding zone. The pattern on fracture surface of the specimen tested at 578 K was quite similar to that of the specimens tested at 207 and 373 K. Usually, the similarity indicates nearly the same fracture toughness [40]; however, the toughness of the sample tested at 578 K was much lower than that of the samples tested at 207 and 373 K. This was unexpected and has not been reported before, as far as we know. The viscous vein pattern on the fracture surface of the sample tested at 578 K might have been caused by the elevated temperature. Therefore, the relationship between toughness and fracture morphology [40] at room temperature cannot be applied to elevated temperatures.

## 4. Discussion

**Figure 5:**
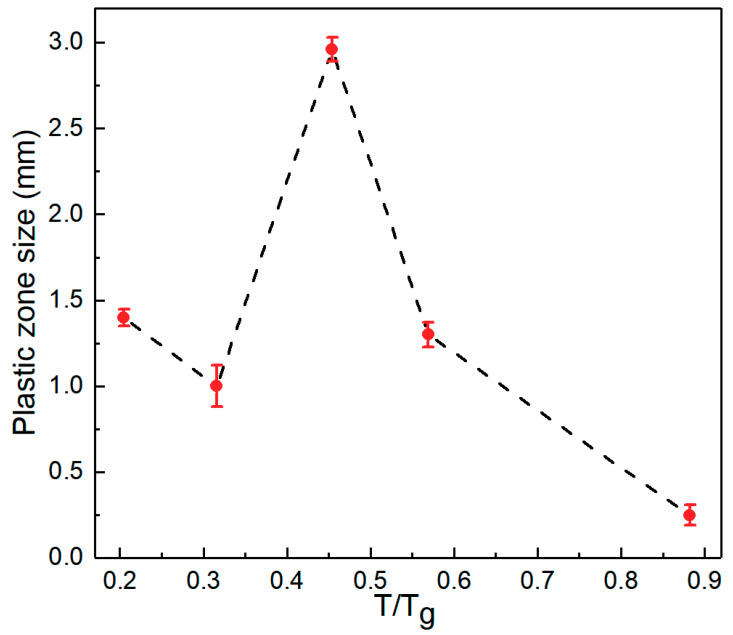
Relationship between plastic zone size of toughness test samples and testing temperature of the Zr_61_Ti_2_Cu_25_Al_12_ BMG.

As described above, shear banding dominates the fracture behavior of Zr_61_Ti_2_Cu_25_Al_12_ BMG. To reveal the mechanism of temperature-dependent fracture toughness of the BMG, the investigation of the physics of flow units that mediate the plasticity of the BMG is necessary. We measured the plastic zone size by measuring the extension of the shear bands in the plastic zone from fatigue pre-crack tip in the direction of loading. Illustrations of the measurement can be found in our previous work [39]. As seen in Figure 1, the lowest point on the relaxation curve is somewhere around room temperature. Figure 5 shows that the specimen tested at room temperature had the largest plastic zone. Figure 5 also shows that the plastic zone sizes of the specimens tested at the onset temperatures of relaxation processes were larger than those of specimens tested at peak temperatures of relaxation processes on the relaxation spectrum in Figure 1. 

The specimens tested at 623 K underwent homogeneous plastic deformation, which is *α*-relaxation of supercooled liquid [32]. Therefore, shear stress can activate relaxation processes (or associated flow units) at their onset temperatures to percolate into *α*-relaxation (shear banding or homogeneous plastic flow) to increase plastic zone size and, hence, fracture toughness. However, at peak temperatures of relaxations, the associated flow units have already been thermally activated and plasticity seldomly had flow units to mediate. From this viewpoint, the temperature-dependent fracture toughness and plasticity, reported in literatures [18,21], can also be reasonably explained.

## 5. Conclusions

Temperature-dependent fracture toughness of Zr_61_Ti_2_Cu_25_Al_12_ BMG with low Hf content was measured from 134 to 623 K. The BMG had the highest toughness at room temperature, relatively lower toughness below room temperature, and intermediate temperature brittleness as reported in the literature [21]. We failed to obtain toughness data of the sample tested at 623 K because the sample did not fracture due to homogeneous viscous flow. The fracture toughness of the BMG was superior to most engineering materials at both cryogenic temperatures and elevated temperatures. Hence, the material has potential applications in engineering structures. Correlations between fracture toughness, fracture morphology, and relaxation spectrum were also investigated. Samples tested at all temperatures exhibited viscous vein patterns on their fracture surface. Only the sample tested at 134 K had dimples on its fracture surface. Shear sliding zones was found on the fracture surface of samples tested at 207, 373, and 578 K. The fracture morphology of the sample tested at room temperature only exhibited viscous vein pattern. We found that the fracture toughness of the BMG was higher at the onset temperatures of relaxation processes than at peak temperatures of the relaxation processes, including fast *β*-relaxation, slow *β*-relaxation, and *α*-relaxation. These relaxation processes correspond to different flow unites [19,30,31,32]. At the onset temperatures, shear stress can activate corresponding relaxations to mediate plasticity while there were rarely flow units to be triggered to percolate into shear banding or homogeneous plastic flow at peak temperatures of relaxation processes. Our viewpoint is helpful for understanding the temperature-dependent fracture toughness and plasticity of BMGs.
